# SARS-CoV-2 detection via metagenomic next-generation sequencing of bronchoalveolar lavage fluid/sputum in lymphoma patients receiving B-cell-depleting therapy: a case report of two cases

**DOI:** 10.3389/fmed.2025.1434340

**Published:** 2025-02-13

**Authors:** Cuiming Sun, Zhidan Zhang

**Affiliations:** Second Department of Infectious Diseases, The First Hospital of China Medical University, Shenyang, Liaoning, China

**Keywords:** SARS-CoV-2, COVID-19, immunocompromised, lymphoma, BALF, mNGS

## Abstract

We describe two cases of coronavirus disease 2019 (COVID-19) infection in patients with lymphoma receiving B-cell-depleting therapy. Metagenomic next-generation sequencing (mNGS) of bronchoalveolar lavage fluid (BALF)/sputum showed ongoing viral replication, despite repeated nasopharyngeal swabs being negative for severe acute respiratory syndrome coronavirus 2 (SARS-CoV-2) RNA. The patients failed to develop seroconversion of IgG antibodies for SARS-CoV-2. However, they showed favorable clinical outcomes after treatment with nirmatrelvir/ritonavir or molnupiravir, despite the antiviral therapies being initiated later in the clinical course. Our case highlights that in immunocompromised hosts, viral clearance of SARS-CoV-2 in lung tissue may lag behind that in the upper respiratory tract. Thus, alternative diagnostic criteria are necessary, and clinical decisions and interventions should be tailored to each individual case.

## 1 Introduction

Individuals with lymphoid malignancy, especially those who receive B-cell-depleting therapies such as anti-CD20 treatments, are prone to protracted coronavirus disease 2019 (COVID-19)-related respiratory symptoms. The risk of severe COVID-19 infection or death rate increases up to 25–34% in these individuals (1, 2). In non-immunocompromised populations, viral replication of severe acute respiratory syndrome coronavirus 2 (SARS-CoV-2) declines approximately 10 days after infection with IgG seroconversion (3). Patients with lymphoma are unable to attain adequate viral clearance, and anti-CD20 treatment within 1 year is an independent predictor for prolonged SARS-CoV-2 detection due to impaired humoral immune response to infection (4). We describe two cases of COVID-19 infection in patients with lymphoma receiving B-cell-depleting maintenance therapy, in whom metagenomic next-generation sequencing (mNGS) of bronchoalveolar lavage fluid (BALF)/sputum revealed persistent viral replication, despite nasopharyngeal swabs testing negative for SARS-CoV-2 RNA.

## 2 Case presentation

### 2.1 Case 1

**Figure 1:**
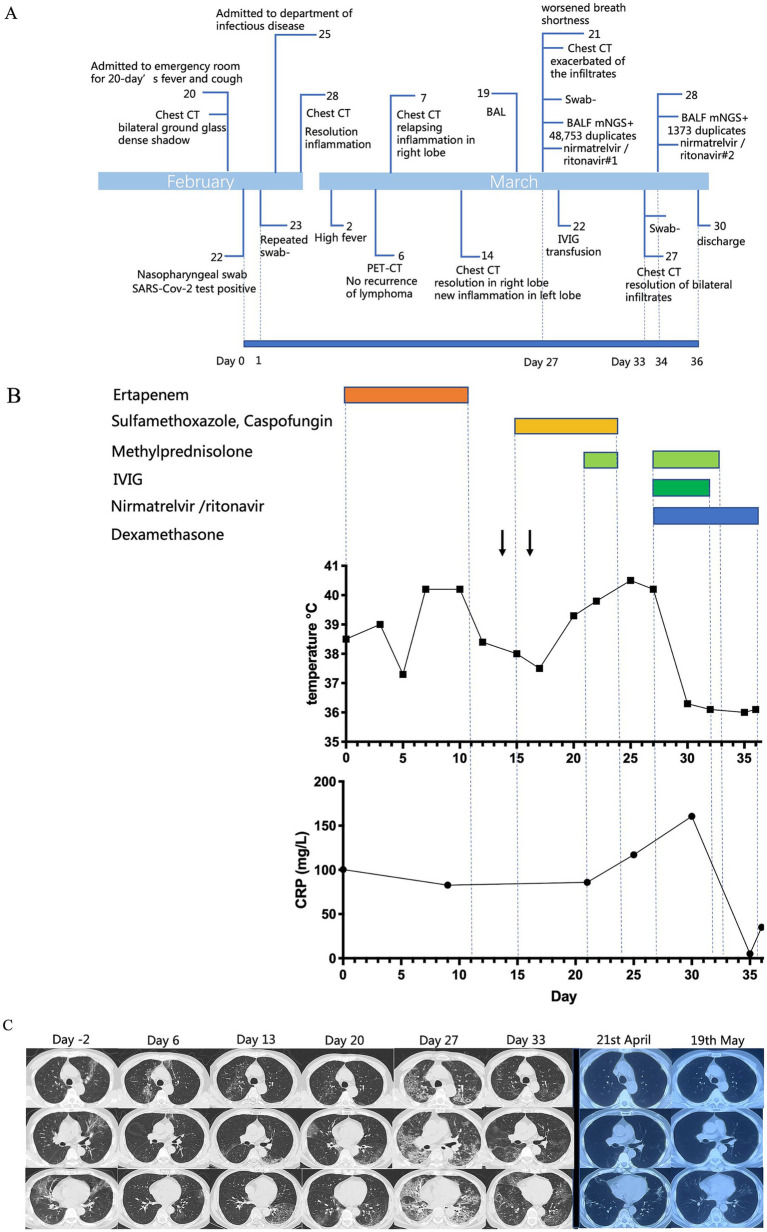
**(A)** Timeline of the case. **(B)** Patient’s body temperature, C-reactive protein (mg/L) plotted by day as the nasopharyngeal swab was positive for SARS-CoV-2, aligned with clinical interventions. **(C)** Chest CT images obtained at indicated time points. From day −2 to day 20, the lung lesions migrated. On day 27, the infiltrates were exacerbated with extensive ground-glass opacification in bilateral lung lobes. Completing the first course of nirmatrelvir/ritonavir treatment, there was a significant resolution of bilateral infiltrates.

The patient was a 63-year-old Chinese man, who was diagnosed with stage III-A follicular non-Hodgkin’s lymphoma 2 years before the current presentation. He received chemotherapy with rituximab (375 mg/m^2^) and epirubicin (50 mg/m^2^) once every 21 days, completing six cycles. The treatment ended in July 2021, and positron emission tomography CT (PET-CT) showed that there was no regrowth of the primary tumor. From then, maintenance treatment with monotherapy of rituximab (375 mg/m^2^) was started. The patient first received a dose of rituximab every quarter for a year, and received doses every six months thereafter. His last rituximab treatment was in September 2022, and he was scheduled to receive the next dose six months later. In February 2023, he was admitted to the emergency room with a 20-day history of fever and dry cough, experiencing a maximum body temperature of over 39°C. On 22nd February (day 0), a SARS-CoV-2 real-time reverse transcription PCR (rt-PCR) test from a nasopharyngeal swab was performed and returned positive (gene N Ct 33.9; gene ORF01ab Ct 34.9). A chest CT scan showed a bilateral ground-glass dense shadow, consistent with viral pneumonia (Figure 1C). Because the Ct values were relatively high, a repeat rt-PCR for SARS-CoV-2 was performed on nasopharyngeal swabs the next day. The result returned negative (day 1), and as a result, he did not receive anti-SARS-CoV-2 treatment. Blood tests showed the following: elevated C reactive protein (CRP) (91.1 mg/L, normal range: 0–6 mg/L), procalcitonin (PCT) (0.153 ng/mL, reference range < 0.046 ng/mL), interleukins and interferon-γ (interleukin-6 14.86 pg/mL, reference range < 5.3 pg/mL; interleukin-10 5.22 pg/mL, reference range < 4.91 pg/mL; and interferon-γ 14.57 pg/mL, reference range < 14.57 pg/mL). His blood routine showed normal white blood cell and neutrophil cell counts with low lymphocyte count (0.88 × 10^9^/L, normal range 1.1–3.2 × 10^9^/L), and concomitant with decreased CD4^+^ T cell count (246 cells/μL, normal range 410–1,590 cells/μL). He was treated with quinolone and cephalosporin for over half a month before receiving further administration. Considering his immunosuppressed state and previous antibiotic therapy, he was treated with ertapenem for suspected community-acquired pneumonia (CAP). Over the following 5 days, the patient steadily recovered, with body temperature decreasing to approximately 37.5°C, and shortness of breath also relieved. The chest CT scan (28th February, day 6) showed resolution of most of the bilateral inflammation. Unexpectedly, 2 days later he developed a high fever again (*T*_max_ 40.2°C). To exclude the recurrence of lymphoma, a PET-CT scan was performed. The standard uptake value of the lymph nodes (cervical, supraclavicular, axillary, mediastinal, and inguinal lymph nodes) was normal. According to the PET-CT results and the consultation of a hematologist, recurrence of lymphoma was ruled out. However, the PET-CT revealed increased bilateral inflammation. On 7th March (day 13), the chest CT scan showed relapsing inflammation in the right lobe. Given the persistent high fever, 5 mg of dexamethasone was intravenously administered when his body temperature exceeded 38.5°C to suppress the inflammatory reaction. After dexamethasone administration, his body temperature remained around 37°C for approximately 36 h. His CD4^+^ T cell count decreased to 238 cells/μL at that time. Due to of the low CD4^+^ T-cell count and the atypical disease course with constant migration of the lung lesions, pneumocystis jirovecii pneumonia (PJP) and cryptogenic organizing pneumonia (COP) were suspected. To ensure the diagnosis, bronchoalveolar lavage (BAL) was recommended but the patient declined. Empirical therapy with compound sulfamethoxazole (for suspected PJP), caspofungin (for suspected PJP), and methylprednisolone (for suspected COP) was initiated after that. He was still febrile. On 14th March (day 20), the chest CT scan showed decreased inflammation in the right lobe, but new inflammation in the left lobe. Relapsing symptoms of high fever and exacerbated pulmonary inflammation prompted us to perform BAL, which was done on 17th March (day 23). On 21st March (day 27), he complained of worsened shortness of breath, and his oxygen saturation dropped to 90%, despite 3 L/min oxygen supplementation. Blood gas analysis revealed type I respiratory failure with an oxygenation index of 241 mmHg. High-flow nasal oxygen was administered, and a chest CT scan was immediately rechecked, which revealed significant exacerbation of the infiltrates. The dramatic deterioration of the symptoms was accompanied by a progressive increase in CRP (160.5 mg/L), PCT (7.1 ng/mL), and reduced CD4^+^ T cell count (141 cells/μL). His SARS-CoV-2 RNA in the nasopharyngeal swab remained negative (day 27). Sputum and blood cultures were tested three times, with all results negative. However, mNGS of the BALF revealed positive SARS-CoV-2 (BA.5.2.48) RNA with 48,753 reads and negative results for other respiratory pathogens. He was diagnosed with SARS-CoV-2 infection. Due to the hypothesis of persistent replication of SARS-CoV-2 in the immunocompromised host, nirmatrelvir/ritonavir (for 5 days) was initiated. Serological testing did not detect antibodies against SARS-CoV-2 (neither IgM nor IgG antibodies). Intravenous immunoglobin (IVIG) was administered at 10 g/day for 5 days. He also received methylprednisolone with a slow tapering regime (80 mg/day for 3 days; 40 mg/day for 2 days). Prone ventilation was used. The patient presented favorable clinical improvement thereafter, with dyspnea improving within 48 h of the combined treatment. A new CT scan (27th March, day 33) showed significant resolution of bilateral infiltrates. He was gradually weaned off oxygen, with oxygen saturation remaining above 95% on room air. Since his rt-PCR for SARS-CoV-2 in the nasopharyngeal swab was negative, the mNGS of BALF was rechecked to evaluate the efficacy of antiviral treatment. Despite severe immunosuppression, duplicates of SARS-CoV-2 RNA in BALF significantly reduced to 1,373 reads (28th March, day 34). Seroconversion was negligible, with a relatively low titer of IgG antibodies for SARS-CoV-2 (5.439 S/CO). Considering the patient was in immunosuppression condition, a further 5-day course of nirmatrelvir/ritonavir was recommended to clear the virus thoroughly. On 30th March (day 36), the patient was free of COVID-19 symptoms and was discharged. He did not have any significant complaints at a follow-up telemedicine visit and returned to normal daily life. We summarize the treatments received, clinical manifestations, and diagnostic tests in Figure 1.

### 2.2 Case 2

**Figure 2:**
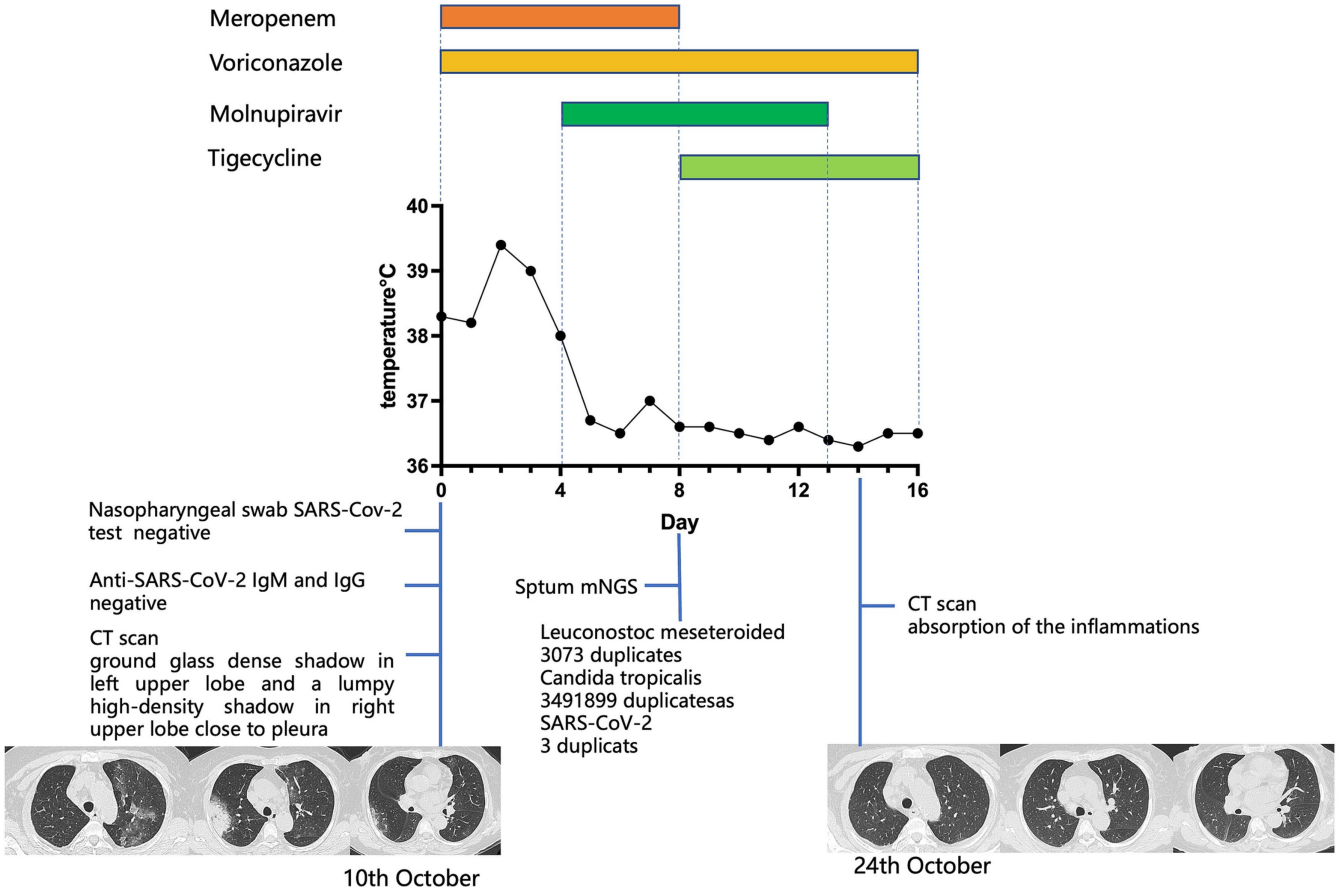
For case 2, the patient’s body temperature, aligned with clinical interventions and the chest CT images obtained at the indicated time points. On day 0, a CT scan showed a ground-glass dense shadow in the left upper lobe and a lumpy high-density shadow in the right upper lobe close to the pleura. On day 14, a CT scan showed absorption of the inflammation.

A 74-year-old Chinese woman was diagnosed with stage IV-a non-Hodgkin’s lymphoma (mantle cell lymphoma, MCL) one and a half years before the current presentation. She received chemotherapy with rituximab (375 mg/m^2^) and epirubicin (50 mg/m^2^) once every 21 days, completing 6 cycles. In November 2022, a PET-CT scan showed local progression. Due to advanced age, she was treated with rituximab and ibrutinib. Half a month before admission, she developed a fever with a *T*_max_ of 38.5°C, cough, and muscular soreness. She self-administered levofloxacin, but it was ineffective, and shortness of breath gradually occurred. She was admitted to our department on 10 October 2023. Blood tests showed the following: elevation of C-reactive protein (CRP) (77.8 mg/L, normal range: 0–6 mg/L), procalcitonin (PCT) (0.076 ng/mL, reference range < 0.046 ng/mL), interleukins and interferon-γ (interleukin-6 52.19 pg/mL, reference range < 5.3 pg/mL; interleukin-10 9.19 pg/mL, reference range < 4.91 pg/mL; and interferon-γ 7.5 pg/mL, reference range < 14.57 pg/mL). Her CD4^+^ T cell count (264 cells/μL, normal range 410–1,590 cells/μL) and B cell count (<1 cell/μL, normal range 90–660 cells/μL) were decreased. SARS-CoV-2 rt-PCR from a nasopharyngeal swab was negative. Both IgM and IgG antibodies for SARS-CoV-2 were undetectable (IgM 0.024, IgG 0.582, reference range 0–1 S/CO). Chest CT scan showed a ground-glass dense shadow in the left upper lobe and a lumpy high-density shadow in the right upper lobe close to the pleura. Considering her immunocompromised status, empirical antibacterial treatment with meropenem, antifungal treatment with voriconazole, and anti-PJP treatment with SMZ were initiated. However, after 3 days of treatment, she remained febrile, with *T*_max_ increasing over 39°C. Upon further inquiry into her medical history, she revealed that her husband had been infected with SARS-CoV-2 half a month before she got sick. Based on the experience and lessons from case 1, we believed that SARS-CoV-2 infection could not be excluded, despite her SARS-CoV-2 rt-PCR from a nasopharyngeal swab and antibodies for SARS-CoV-2 being negative. Molnupiravir was administered immediately with consent from the patient and her family. The day after starting molnupiravir, her body temperature returned to normal. Subsequent mNGS results from sputum showed *Leuconostoc meseteroided* (3,073 reads), *Candida tropicalis* (3,491,899 reads), and SARS-CoV-2 (3 reads), confirming our previous assumption. According to the results, meropenem was replaced by tigecycline, and the administration of voriconazole and molnupiravir was continued. Two weeks later, her chest CT scan showed apparent absorption of the inflammation, and the patient was totally relieved from fever and dyspnea. We summarize the treatments received, clinical manifestations, and diagnostic tests in Figure 2.

## 3 Discussion and conclusion

In this report, we described two cases of long-lasting COVID-19 infection patients with non-Hodgkin’s lymphoma receiving rituximab. The clinical manifestation of case 1 was characterized by relapsing high fever and migrated lower airway changes on the chest CT scan. Both case 1 and case 2 exhibited persistent RNA replication in the lungs, as indicated by mNGS of BALF, while the SARS-CoV-2 virus in nasopharyngeal swabs had already been cleared and remained negative for a long period.

The patients’ lymphoma, predisposed condition of previous anti-CD20 therapy, and associated immunosuppression are likely the reasons for the long-lasting clinical course. Anti-CD20 monoclonal antibodies, such as rituximab, induce rapid depletion of more than 95% of CD20-positive mature B cells, affecting both malignant B cells and normal B cells, thereby impairing cellular and humoral responses to infections (5). Treatment with anti-CD20 antibodies within 1 year is one of the independent predictors of prolonged viral replication and shedding of SARS-CoV-2 (4). Patients with lymphoma, especially those with a diminished T-cell response after B-cell-depleting therapy, are at a higher risk for a prolonged disease course and poor outcomes after acute COVID-19 infection. Patients with severe CD8 lymphopenia (<50 cells/μL) or CD4 lymphopenia (<100 cells/μL) had markedly poor outcomes (4). Meanwhile, the defective humoral response due to B-cell-depleting treatment leads to the absence of effective seroconversion and recrudescence of inflammation. Studies have revealed that patients with lymphoma undergoing rituximab therapy who fail to develop anti-SARS-CoV-2 antibodies are at high risk of having severe and prolonged COVID-19 infection (6). There are several case reports about the prolonged viral shedding duration in patients with lymphoma demonstrated by persistent SARS-CoV-2 PCR replication in their nasopharyngeal or sputum samples (5–7). However, in our case, repeated rt-PCR for SARS-CoV-2 in nasopharyngeal swab remained negative, while mNGS of BALF revealed persistent viral replication. This suggests that viral clearance in the lower respiratory tract may lag behind that in the nasopharynx, and immunocompromised individuals may act as a reservoir of SARS-CoV-2. Until now, the vast majority of testing is still carried out using PCR-based systems, with sensitivity at 63% for nasal swabs (7). Sampling techniques and nucleic acid extraction are potential sources of error. NGS platforms are accurate, reliable and widely used for the detection of viral presence. However, this method is expertise, more prolonged, and laborious, it is not recommended as the diagnostic criteria for COVID-19 infection in the Chinese COVID-19 treatment guidelines. In this case, mNGS of BALF provided strong evidence for COVID-19 infection. Our case highlights that in immunocompromised hosts, SARS-CoV-2 RNA can be detected in lung tissue long after nasopharyngeal swabs become negative, suggesting that these patients may require alternative diagnostic criteria.

Nirmatrelvir/ritonavir reduced hospital admission or death by approximately 90% among patients receiving treatments within 3 days of COVID-19 infection (8). Compared to nirmatrelvir/ritonavir, molnupiravir seldom interacts with other drugs. Considering the combined treatment with voriconazole, the second patient was administered molnupiravir. According to the Chinese COVID-19 diagnosis and treatment guidelines, nirmatrelvir/ritonavir and molnupiravir were recommended to the patients within 5 days of onset. Although nirmatrelvir/ritonavir and molnupiravir were administered later in the patient’s clinical course, the patients still exhibited a significant clinical response closely correlated with the administration of these antiviral drugs. This suggests that nirmatrelvir/ritonavir and molnupiravir can be effective even when administered later in the course of infection. IVIG has previously been successfully used in several inflammatory and autoimmune diseases; however, the potential beneficial effect in COVID-19 patients was controversial. Similar to cases in which IVIG was used for treatment (9), case 1 suggested that IVIG contributes to the control of SARS-CoV-2, at least in some patients, probably due to the blockade of Fc receptors or its neutralization activity against SARS-CoV-2 (10). Being a case report, there are a few limitations of our study. We did not perform rt-PCR of SARS-CoV-2 in BALF. As a result, we were not able to determine the changes in viral copy numbers to evaluate the efficacy of nirmatrelvir/ritonavir and molnupiravir accurately.

Due to the persistent negative SARS-CoV-2 result from the nasopharyngeal swab, the diagnosis was delayed in case 1. However, with the lessons learned from case 1, anti-viral treatment was administered in a timely manner in case 2, significantly shortening the clinical course. Although this report focused on long-lasting COVID-19 in immunocompromised individuals with lymphoma receiving anti-CD20 treatment, other forms of immunodeficiency, such as in individuals with solid cancers, HIV infection, hematopoietic stem cell transplant recipients, and those receiving chemotherapy and corticosteroids, also create opportunities for prolonged SARS-CoV-2 viral replication. Strategies to avoid unfavorable outcomes should be carefully considered in immunocompromised conditions.

Taken together, our observation suggests that for immunocompromised patients highly suspected of COVID-19 infection, when nasopharyngeal swabs are negative, additional testing on other specimens such as BALF may be a rational approach. Immunocompromised hosts, who may experience prolonged COVID-19 infection, require alternative diagnostic criteria. It is important to establish individualized decisions and interventions tailored to the distinct clinical characteristics of immunocompromised patients during this historic pandemic.

## Data Availability

The raw data supporting the conclusions of this article will be made available by the authors without undue reservation.
